# Definitive radiotherapy for non-small-cell lung cancer: current practice and future directions

**DOI:** 10.1007/s11604-026-02005-6

**Published:** 2026-05-20

**Authors:** Noriko Kishi, Tomoki Kimura, Takashi Mizowaki

**Affiliations:** 1 Department of Radiation Oncology, Kyoto University Hospital, 54 Shogoinkawahara-cho, Sakyo-ku, Kyoto, 606-8507 Japan; 2 Department of Radiation Oncology, Kochi Medical School, Kochi University, Kohasu, Okou Town, Nankoku City, Kochi 783-8505 Japan

**Keywords:** Non-small cell lung cancer, Radiotherapy, Chemoradiotherapy, Immune checkpoint inhibitor, Oligometastasis

## Abstract

Lung cancer is one of the malignancies for which new clinical evidence has accumulated most rapidly in recent years. In parallel with advances in systemic therapy, radiotherapy has also evolved substantially with the development of high-precision techniques such as stereotactic body radiotherapy (SBRT), intensity-modulated radiotherapy, and particle therapy. These technological innovations, together with the introduction of immunotherapy and targeted therapies, have reshaped the role of radiotherapy within multidisciplinary treatment strategies for non-small cell lung cancer (NSCLC). This review summarizes current clinical practice and emerging perspectives in radiotherapy for NSCLC across disease stages. In early-stage disease, the established role of SBRT and the potential advantages of particle therapy as curative strategies for medically inoperable patients and for operable patients who refuse surgery are outlined. For locally advanced NSCLC, evolving treatment paradigms in the era of immunotherapy are reviewed, including concurrent chemoradiotherapy followed by consolidation immunotherapy, as well as ongoing efforts to reduce treatment-related toxicity through advances in radiotherapy techniques. In addition, the emerging role of radiotherapy as local consolidative therapy for oligometastatic stage IV NSCLC is highlighted, with attention to recent randomized trials and the challenges of patient selection. As treatment strategies for NSCLC continue to evolve rapidly, radiotherapy is expected to play an increasingly important role in multidisciplinary management. Further research is required to optimize treatment sequencing, identify patients most likely to benefit from radiotherapy, and refine its integration with modern systemic therapies. Radiotherapy remains a key component of multidisciplinary management for non-small cell lung cancer (NSCLC). This review summarizes current clinical evidence and the evolving roles of radiotherapy across disease stages, including early-stage, locally advanced, and oligometastatic NSCLC.

## Introduction

Lung cancer is one of the most common malignancies worldwide, accounting for a substantial proportion of cancer incidence and mortality [1]. Compared with many other malignancies, lung cancer management has been reshaped by the identification of molecular subtypes, enabling increasingly refined, biomarker-driven, and personalized treatment strategies. In systemic therapy, treatment options have expanded rapidly beyond conventional cytotoxic chemotherapy to include immune checkpoint inhibitors (ICIs) and an expanding range of targeted therapies. Surgery has also evolved toward less invasive approaches, not only through advances in procedures—from open thoracotomy to video-assisted thoracoscopic surgery and robotic-assisted surgery—but also through a shift toward the smaller extent of resection.

Radiotherapy has advanced toward high-precision techniques such as intensity-modulated radiotherapy (IMRT) and stereotactic body radiotherapy (SBRT), and its role and optimal integration with systemic therapy and surgical procedures continue to evolve in the context of the exceptionally rapid progress in lung cancer management. In this review, we summarize the established evidence supporting current treatment strategies for lung cancer, with a focus on non-small cell lung cancer (NSCLC), and discuss future directions in which radiotherapy is expected to contribute to multidisciplinary care.

## Stage I–II NSCLC

### Current clinical practice

For stage I NSCLC, the extent of resection is increasingly individualized according to tumor size and the consolidation-to-tumor (C/T) ratio, particularly for peripherally located stage IA1–2 tumors ≤ 2 cm, based on randomized trials. In brief, a C/T ratio ≤ 0.25 generally supports sublobar resection (segmentectomy or wedge resection), 0.25–0.5 supports segmentectomy, and > 0.5 supports segmentectomy or lobectomy depending on patient- and tumor-related factors [2–5]. For patients who are not candidates for such standard surgery, sublobar resection or definitive radiotherapy are considered treatment options. For stage II NSCLC, lobectomy with systematic lymph node dissection is generally recommended for patients who are medically operable [6], whereas definitive radiotherapy or concurrent chemoradiotherapy is recommended for patients who are medically inoperable, depending on their general conditions and comorbidities. Because nodal recurrence in the hilar or mediastinal lymph nodes after definitive radiotherapy directed at the stage I–II NSCLC is relatively uncommon [7], high-precision radiotherapy for localized primary tumor—namely SBRT and particle therapy (proton and carbon-ion radiotherapy)—is widely used.

### SBRT in peripherally located stage I–II NSCLC

SBRT was developed primarily in Japan, the United States, and Sweden from the late 1980s to the early 1990s. It delivers highly conformal, ablative doses over a short treatment period, typically within 1 or 2 weeks, while minimizing the dose delivered to surrounding normal tissues. After SBRT techniques were established, multiple phase II trials were conducted mainly in the 2000s in patients with peripherally located NSCLC who were medically inoperable or who refused surgery. Representative trials are summarized in Table 1. These studies reported local control rates approaching 90%, with grade 4 or higher severe adverse events occurring in less than 5% of patients, although SBRT in most of these studies was delivered using three-dimensional conformal radiotherapy (3D-CRT) rather than IMRT. Based on Radiation Therapy Oncology Group (RTOG) 0236 and RTOG 0618, a three-fraction regimen has been widely adopted in the United States, whereas in Japan a four-fraction regimen established by Japan Clinical Oncology Group (JCOG) 0403 has been commonly used [8–11]. Of note, 3-year overall survival (OS) rates after lobectomy for T1–2 NSCLC have historically been as high as 90% in cohorts with a median age of 66–67 years [12], whereas the median ages in RTOG 0236 and RTOG 0618 were 72–73 years; moreover, the JCOG trials of SBRT enrolled much older populations with median age of 78–81 years. Collectively, SBRT has demonstrated excellent local control with acceptable toxicity, even in elderly patients.

**Table 1:** Representative phase II trials of stereotactic body radiotherapy (SBRT) for peripherally located stage I–II non-small cell lung cancer (NSCLC)

Trial/author (year)	*N*	T category	Operability	Prescribed dose	3-y OS (%)	3-y LC (%)	AEs ≥ G3 (%)		
							G3	G4	G5
Baumann P, et al. (2009) [8]	57	T1–2	Inoperable or refusal of surgery	45 Gy/3 fr. prescribed to the periphery of the PTV (= 66 Gy/3 fr. to IC)	60	92	28	1.8	0.0
RTOG 0236 (2010) [9]	55	T1–2 (< 5 cm)	Inoperable	54 Gy/3 fr. prescribed to the periphery of the PTV^*^	55.8	97.6	12.7	3.6	0.0
JCOG 0403 (2015) [10]	100	T1	Inoperable	48 Gy/4 fr. to IC	59.9	87.3	10.0	2.0	0.0
	64		Operable^†^		76.5	85.4	7.8	0.0	0.0
RTOG 0618 (2018) [11]	26	T1–2 (< 5 cm)	Operable^‡^	54 Gy/3 fr. prescribed to the periphery of the PTV	56 (4-y)	96 (4-y)	15.3	0.0	0.0

AE, Adverse events; fr., Fractions; G, Grade; IC, Isocenter; JCOG, Japan Clinical Oncology Group; LC, Local control; N, Number of patients; OS, Overall survival; PTV, Planning target volume; RTOG, Radiation Therapy Oncology Group

*Actual delivered dose calculated with appropriate heterogeneity correction

^†^Classified by surgeons

^‡^At least suitable for sublobar resection

JCOG 0702 was a phase I dose-escalation study that enrolled 28 patients with peripheral T2 tumors more than 3 cm in diameter who were either unfit for lobectomy or who refused surgery and were aged ≥ 70 years [13]. The protocol planned dose escalation from 40 Gy in 4 fractions to 65 Gy in 4 fractions in 5-Gy increments to determine the tolerated dose. The recommended dose was 55 Gy for patients with planning target volume (PTV) < 100 cm^3^ and 50 Gy for those with PTV ≥ 100 cm^3^. JCOG 1408, a phase III trial comparing the regimen used in JCOG 0403 with that in JCOG 0702 [14], is currently ongoing to determine whether dose escalation improves clinical outcomes, and its results are awaited.

### Key clinical evidence comparing SBRT with surgery

Given the expectation that SBRT could serve as an alternative local curative therapy to surgery, several phase III randomized trials comparing SBRT with lobectomy or sublobar resection in patients with operable early-stage NSCLC were initiated, but terminated early because of poor accrual. Among these, a pooled analysis of the STARS and ROSEL trials has been reported [15]. The pooled population comprised 58 operable patients with tumors ≤ 4 cm and a median age of 67 years. Three-year OS was 95% in the SBRT group and 79% in the lobectomy group, favoring SBRT (hazard ratio [HR], 0.14; 95% confidence interval [CI], 0.017–1.190; *p* = 0.037). Three-year local recurrence–free survival was 96% with SBRT and 100% with lobectomy, with no statistically significant difference between the two groups (*p* = 0.44). No grade 4 or higher adverse events were reported after SBRT, whereas 4% of patients in the surgery group died from complications. However, several limitations have been highlighted, including the small sample size, the non-prespecified nature of the pooled analysis, and heterogeneity between the two trials. Accordingly, current clinical guidelines consider the evidence insufficient to recommend SBRT as equivalent to surgery for operable patients.

The SABRTooth trial, a randomized feasibility study in peripheral stage I NSCLC among patients at high surgical risk, concluded that a phase III trial was not feasible in the National Health Service settings [16]. It evaluated recruitment feasibility and the target monthly recruitment rate was 3 patients, whereas the observed rate was only 1.7 patients per month. The most common reason for non-participation was treatment preference. In contrast, in the United States, phase III trials are ongoing and accruing, including VALOR (SBRT vs. lobectomy/segmentectomy; target accrual, 670; study completion anticipated in 2034; NCT02984761) and STABLE-MATES (SBRT vs. segmentectomy/wedge resection; target accrual, 272; study completion anticipated in 2028; NCT02468024), and their results are awaited.

Although robust evidence from fully accrued randomized trials directly comparing SBRT with surgery has not yet been established, SBRT has been shown to provide excellent local control with low toxicity. Therefore, from the perspective of shared decision-making, patients should be informed about SBRT as a potential alternative treatment option.

### Centrally located stage I–II NSCLC

SBRT for centrally located lung tumors is associated with a higher risk of severe toxicity than SBRT for peripherally located lung tumors, and the optimal dose fractionation remains an important clinical issue [17]. A commonly used definition of “central” is a tumor located within 2 cm of the proximal bronchial tree (PBT), as in RTOG 0236 [9]. The PBT typically includes the distal trachea (within 2 cm above the carina), the main bronchi, and the lobar bronchi. In addition, an IASLC definition has been used that incorporates proximity within 2 cm to mediastinal critical structures such as the esophagus, heart, brachial plexus, great vessels, spinal cord, phrenic nerve, and recurrent laryngeal nerve [18]. Gross tumor volume (GTV) or planning target volume (PTV) adjacent to the PBT, esophagus, or major vessels are often classified as “ultra-central,” for which a more fractionated regimen is generally preferred. Representative phase I–II studies of SBRT for central and ultra-central lung tumors are summarized in Table 2. Regimens of 5 or 8 fractions are commonly used, with reported 3-year local control (LC) rates of approximately 90% [19–23]. The LUSTRE trial, a phase III study, evaluated whether SBRT at 48 Gy in 4 fractions for peripherally located lesions or 60 Gy in 8 fractions for centrally located lesions improves LC compared with hypofractionated conventional radiotherapy (CRT) using 60 Gy in 15 fractions in patients with medically inoperable stage I (≤ 5 cm) NSCLC [24]. Although the trial was closed early because of poor accrual, no significant difference in LC was observed between the groups, and severe adverse events were limited.

**Table 2:** Representative trials of radiotherapy for centrally located stage I–II NSCLC or metastatic pulmonary tumors

Trial (year)	Phase	*N*	Eligibility	Prescribed dose	3-y OS (%)	3-y LC (%)	AEs ≥ G3 (%)		
							G3	G4	G5
JROSG 10-1 (2017) [19]	I	9	≤ 3 cm; tumor within or touching the PBT or adjacent to the mediastinal or pericardial pleura	SBRT 60 Gy/8 fr. to IC	76.2	–	0.0	0.0	0.0
RTOG 0813 (2019) [20]	I/II	33	≤ 5 cm; tumor located ≤ 2 cm from the PBT or adjacent to the mediastinal or pericardial pleura	SBRT 60 Gy/5 fr. (D_99%_ ≥ 54 Gy)	72.7 (2-y)	87.9	15.2	3.0	3.0
		38		SBRT 57.5 Gy/5 fr. (D_99%_ ≥ 51.75 Gy)	67.9	89.4	13.1	0.0	7.9
HILUS (2021) [21]	II	65	Primary or metastatic lung cancer (≤ 5 cm); tumor located ≤ 1 cm from the PBT	SBRT 56 Gy/8 fr. prescribed to the periphery of the PTV	50	83	18.5	15.4	
LungTech (2024) [22]	II	39	≤ 7 cm; tumor located ≤ 2 cm from the PBT or adjacent to the mediastinal or pericardial pleura	SBRT 60 Gy/8 fr. (D_99%_ ≥ 54 Gy)	61.6	81.5	Acute: 3.2Late: 16.1	Acute: 3.2 Late: 3.2	
SUNSET (2024) [23]	I	30	≤ 6 cm; PTV touching or overlappings the bronchial tree, esophagus, pulmonary vein, or pulmonary artery	SBRT 60 Gy/8 fr. (D_95%_ prescription; D_99%_ ≥ 54 Gy)	72.5	89.6	3.3	0.0	3.3
LUSTRE (2024) [25]	III	45	≤ 5 cm; tumor located within 1 cm of the mediastinum and/or within 2 cm of the PBT^*^	SBRT 60 Gy/8 fr	63.5^*^	87.6^*^	11.1	2.2	
		19		CRT 60 Gy/15 fr	68.4^†^	81.2^†^	5.3	0.0	

CRT, Hypofractionated conventional radiotherapy; MTD, Maximum tolerated dose; PBT, Proximal bronchial tree. The other abbreviations are the same as Table 1

*Including 109 additional patients with peripherally located NSCLC treated with SBRT 48 Gy in 4 fractions

^†^Including 60 additional patients with peripherally located NSCLC treated with CRT 60 Gy in 15 fractions

Thus, SBRT can be considered a useful treatment option for central and ultra-central lung tumors as well as for peripherally located lung tumors. However, its use requires careful selection of fractionation schedules and strict adherence to organ-at-risk (OAR) dose constraints. JROSG 19-1, a nationwide prospective observational study in Japan investigating high-precision hypofractionated radiotherapy for centrally located NSCLC smaller than 3 cm in diameter, is currently ongoing to clarify real-world treatment outcomes and AEs among three fractionation schedules: 4, 8, and 25 fractions. To further reduce OAR dose, online adaptive radiotherapy has been proposed as a promising approach to improve treatment safety and efficacy. Ongoing studies include the phase I dose-escalation MAGELLAN trial investigating daily adaptive MR-guided SBRT (NCT04925583), the STAR-LUNG study evaluating the feasibility and safety of daily adaptive MR-guided SBRT for ultra-central lung tumors (NCT05354596), and the PUDDING study assessing daily adaptive CBCT-guided SBRT (jRCT1052230085) [25].

### Particle radiotherapy

Particle radiotherapy, including proton and heavy-ion therapy, has been developed as an advanced irradiation technique that utilizes the Bragg peak to achieve a sharp dose fall-off beyond the distal edge of the tumor. This allows high-dose irradiation to the tumor while reducing OAR dose. In particular, heavy-ion therapy, such as carbon-ion therapy, may offer an additional biological advantage because of its high linear energy transfer, which is associated with greater DNA-damaging capability.

In clinical practice, a Japanese nationwide retrospective study including patients with operable or medically inoperable stage I–IIA NSCLC reported outcomes of proton beam therapy. In the medically inoperable cohort, the 3-year OS was 70.5%, the 3-year PFS was 56.6%, and the local progression-free rate was 87.5%, with grade 3 or higher adverse events observed in 2.8% of the overall cohort [26]. A more recent prospective registry study in patients with inoperable stage I–IIA NSCLC reported a 3-year OS of 62.7%, a 3-year PFS of 52.9%, and a local failure rate of 14.4%, with no grade 4 or higher adverse events [27], although 15% of patients had underlying interstitial lung disease and were considered at high risk for radiation pneumonitis.

For carbon-ion radiotherapy, the prospective nationwide registry study J-CROS-LUNG, which included all carbon-ion radiotherapy institutions in Japan, reported outcomes in medically inoperable stage I NSCLC, with a 3-year OS of 59.3% and a local control rate of 87.3% [28]. Grade 3 radiation pneumonitis occurred in 2.1% of patients, and no grade 4 or higher events were observed. In the same registry study focusing on operable stage I NSCLC, the 5-year OS was 81.8%, PFS was 65.9%, and local control was 95.8%, with grade 3 radiation pneumonitis reported in 0.7% of patients and no grade 4 or higher events [29].

Overall, particle therapy for stage I–II NSCLC can achieve high local control rates with a very low incidence of severe toxicity, largely owing to its favorable physical dose distribution, even in cohorts that included patients at high risk for radiation pneumonitis, such as those with interstitial lung disease [30, 31]. Particle therapy may therefore be considered a valuable option in clinical settings where enhanced dose conformity or normal tissue sparing is desirable, although high-level evidence demonstrating the superiority of particle therapy over photon SBRT remains limited.

### Future directions: adjuvant therapy after SBRT

The role of adjuvant therapy after SBRT for stage I–II NSCLC remains uncertain. The benefit of chemotherapy has not been clearly established; however, for tumors ≥ 5 cm, the addition of chemotherapy has been associated with improved OS in retrospective analyses [32]. More recently, ICIs have been investigated in combination with SBRT. The I-SABR trial, a randomized phase II study in stage I–II NSCLC, compared SBRT alone with SBRT plus nivolumab and reported significantly improved 4-year event-free survival (EFS) with the addition of nivolumab (HR, 0.38; 95% CI 0.19–0.74; *p* = 0.0056) [33]. However, the phase III KEYNOTE-867 trial, which evaluated SBRT with or without pembrolizumab with EFS as the primary endpoint, was terminated at interim analysis because of an increased incidence of adverse events, including grade 5 events (NCT03924869). Similarly, the phase III SWOG/NRG S1914 trial, which investigated neoadjuvant and consolidation atezolizumab in combination with SBRT versus SBRT alone, with OS as the primary endpoint, was also discontinued after interim analysis failed to demonstrate benefit (NCT04214262). Another ongoing phase III trial, PACIFIC-4, is evaluating SBRT with or without durvalumab (NCT03833154). In this trial, a dedicated subcohort of patients harboring epidermal growth factor receptor (EGFR) tyrosine kinase inhibitor (TKI)-sensitizing mutations will receive adjuvant osimertinib following SBRT for stage I–II lymph node-negative NSCLC to evaluate its efficacy in this setting. LIBRETTO-432 is another randomized study assessing adjuvant selpercatinib after definitive surgery or radiotherapy in patients with stage IB–IIIA RET fusion-positive NSCLC [34] (NCT04819100).

These studies reflect growing interest in adjuvant systemic therapy following SBRT, particularly in molecularly defined subgroups, paralleling treatment strategies established for locally advanced and metastatic NSCLC. However, a major challenge in the SBRT setting is that histologic confirmation is often unavailable or tissue samples are insufficient for biomarker testing, which limits the implementation of personalized treatment strategies. Consequently, imaging-based biomarkers are increasingly expected to contribute to treatment selection and risk stratification, particularly from the perspective of diagnostic radiologists and radiation oncologists, who often have access to imaging.

## Stage III NSCLC

### Current clinical practice

The standard treatment strategy is determined primarily by both resectability and the feasibility of definitive radiotherapy. “Resectable” stage III NSCLC is generally managed with surgery with or without perioperative therapy, whereas “unresectable” NSCLC is treated with concurrent chemoradiotherapy when definitive radiotherapy is feasible. Definitive radiotherapy alone may be considered when chemotherapy is not feasible because of impaired cardiac or renal function. When definitive radiotherapy cannot be safely delivered, systemic therapy is typically administered according to treatment strategies for stage IV NSCLC.

There is no universally accepted definition of “resectable” stage III NSCLC, and criteria differ substantially across institutions and thoracic surgeons, leading to variability in clinical decision-making [35, 36]. The latest UICC 9th edition introduced updated N2 descriptors, subclassifying N2 disease into N2a (single-station involvement) and N2b (multi-station involvement). However, this classification alone does not adequately define resectability. Other factors, including patterns of nodal involvement (e.g., discrete versus infiltrative disease), the presence of bulky nodal disease, the potential need for pneumonectomy, and the anatomic relationship between the primary tumor and involved lymph nodes, also influence surgical decision-making. As a result, stage III NSCLC represents a highly heterogeneous disease.

### Key clinical evidence of resectable stage III NSCLC

The evidence supporting perioperative radiotherapy or chemoradiotherapy is derived largely from trials conducted in the 1990s and early 2000s, when irradiation techniques were limited to two-dimensional radiotherapy or 3D-CRT rather than IMRT.

INT-0139, a phase III trial comparing definitive chemoradiotherapy with neoadjuvant concurrent chemoradiotherapy followed by surgery in patients with resectable, pathologically confirmed N2 NSCLC, did not demonstrate an OS benefit for the surgical arm [37]. In an exploratory analysis, OS appeared to be improved in patients who underwent neoadjuvant concurrent chemoradiotherapy followed by lobectomy, but not in those requiring pneumonectomy, compared with patients treated with definitive chemoradiotherapy. The WJTOG9903 trial compared neoadjuvant chemotherapy with neoadjuvant concurrent chemoradiotherapy followed by surgery in patients with pathologically confirmed N2 NSCLC but was prematurely terminated because of slow accrual [38]. Although the chemoradiotherapy arm achieved a higher downstaging rate (40.0% vs. 20.8% in the chemotherapy arm), no significant difference in OS was observed (HR, 0.77; *p* = 0.40). Similarly, the SAKK 16/00 trial compared neoadjuvant chemotherapy with neoadjuvant sequential chemoradiotherapy followed by surgery in patients with pathologically confirmed N2 NSCLC [39]. No significant difference in OS was observed (median OS, 37.1 months in the chemoradiotherapy group vs. 26.2 months in the chemotherapy group; HR, 1.0; 95% CI 0.7–1.4). Postoperative radiotherapy was administered in 16% of patients in the chemotherapy arm. The response rate (complete or partial response) was higher in the neoadjuvant sequential chemoradiotherapy group than in the chemotherapy group (61% vs. 44%). Thus, three representative trials—INT-0139, WJTOG9903, and SAKK 16/00—are frequently cited as evidence for neoadjuvant chemoradiotherapy. However, these studies have several limitations: they were conducted using older irradiation techniques, differed in patient populations and intervention strategies, and their results may have been influenced by subsequent treatments. Consequently, none of these trials demonstrated a statistically significant improvement in overall survival. In contrast, recent phase III trials incorporating chemotherapy with ICIs for resectable stage II to N2 stage IIIB NSCLC have demonstrated significant improvements in EFS. These include CheckMate-816 (neoadjuvant nivolumab), KEYNOTE-671 (perioperative pembrolizumab), Neotorch (perioperative toripalimab), AEGEAN (perioperative durvalumab), and CheckMate-77T (perioperative nivolumab) [40–44]. Across these studies, pathological complete response (pCR) rates ranged from 17.2% to 25.3%, exceeding those historically reported with chemoradiotherapy-based approaches (3.5%–9.7%) [38, 39], although pCR is not necessarily a validated surrogate endpoint for OS.

For T3–4 N0–1 superior sulcus NSCLC, a multidisciplinary approach incorporating neoadjuvant chemoradiotherapy followed by surgical resection is recommended, primarily based on phase II trials. In SWOG 9416, which evaluated concurrent chemoradiotherapy followed by surgery, the pCR rate was 36.3% among patients who underwent thoracotomy, with a 5-year OS of 44% [45]. JCOG 9806 also investigated neoadjuvant chemoradiotherapy and reported a pCR rate of 16.0% and a 5-year OS of 56% [46]. Similarly, CJLSG0801, which evaluated chemoradiotherapy followed by surgery for T3N0–1 NSCLC including superior sulcus tumors, demonstrated a pCR rate of 26% and a 5-year OS of 62.6% [47]. More recently, JCOG1807C (DEEP OCEAN), a prospective trial evaluating perioperative durvalumab (before and after surgery) or durvalumab following chemoradiotherapy, is ongoing [48]. For superior sulcus NSCLC, conventional chemoradiotherapy has historically shown relatively favorable pCR rates; therefore, it remains of interest whether the addition of ICIs can further improve clinical outcomes.

Earlier studies suggested that neoadjuvant chemoradiotherapy could be beneficial in selected patients. However, the emergence of ICI-based strategies supported by recent phase III trials has reduced the relative role of chemoradiotherapy in current treatment paradigms. Future research should therefore focus on identifying patient subsets that may benefit from radiotherapy combined with ICIs, incorporating high-precision irradiation techniques and molecularly guided treatment strategies.

### Key clinical evidence of unresectable stage III NSCLC

Evidence regarding the optimal fractionation for unresectable stage III NSCLC is derived largely from older studies. In particular, the RTOG 7301 trial conducted in the 1970s established the current standard dose of 60 Gy [49]. This study compared four fractionation schedules: a 40 Gy split-course regimen and continuous regimens of 40, 50, or 60 Gy. The 60 Gy continuous regimen demonstrated the lowest incidence of local failure and was therefore adopted as the standard dose. However, the trial also highlighted that distant metastasis remained frequent, indicating the need for effective systemic therapy. Subsequent studies demonstrated that the addition of chemotherapy to radiotherapy improves OS, with concurrent chemotherapy providing greater benefit than sequential chemoradiotherapy [50]. Several trials were then conducted to further improve OS by optimizing concurrent chemotherapy regimens. However, comparisons between second-generation and third-generation chemotherapy regimens did not demonstrate a significant improvement in OS [51]. On the radiotherapy side, dose escalation was also investigated with the expectation that higher radiation doses might improve survival. The RTOG 0617 trial, a randomized phase III study comparing 74 Gy with the standard 60 Gy using either 3D-CRT or IMRT, unexpectedly showed worse OS in the high-dose cohort, and the strategy of dose escalation therefore failed to improve outcomes [52].

In 2017, the PACIFIC trial marked a major breakthrough in the treatment strategy for unresectable stage III NSCLC [53]. This randomized phase III study compared maintenance durvalumab with placebo for up to 12 months in patients who had received concurrent platinum-based chemoradiotherapy (≥ 54 Gy) without disease progression. Durvalumab significantly improved OS; the 5-year OS was 42.9% in the durvalumab group compared with 33.4% in the placebo group. Regarding toxicity, the incidence of grade 3 or 4 adverse events of any cause was similar between the two groups (30.5% in the durvalumab group vs. 26.1% in the placebo group). However, the magnitude of benefit from durvalumab has been questioned in certain subgroups, particularly patients with low PD-L1 expression or those harboring driver mutations [54, 55].

In patients with unresectable EGFR-mutated stage III NSCLC who had not experienced progression during or after chemoradiotherapy, the phase III LAURA trial, comparing osimertinib with placebo administered until disease progression, has been conducted [56]. The primary endpoint was PFS, which was significantly improved in the osimertinib group compared with the placebo group (39.1 months vs. 5.6 months; HR, 0.16; 95% CI 0.10–0.24; *p* < 0.001). An interim analysis showed no statistically significant difference in OS (HR, 0.81; 95% CI 0.42–1.56; *p* = 0.53), although OS data remain immature. As the study protocol allowed crossover to osimertinib upon disease progression in the placebo group, mature OS results are awaited.

### Future directions: reducing radiotherapy-induced toxicity

After the PACIFIC trial, pneumonitis has been recognized as one of the most frequent adverse events leading to discontinuation of the PACIFIC regimen [53]. Similarly, in the LAURA trial, the most common adverse event was radiation pneumonitis, with grade 3 or higher pneumonitis occurring in 2% of patients in the osimertinib group and 0% in the placebo group [56]. Because the administration and continuation of maintenance systemic therapy may be affected by treatment-related pulmonary toxicity, minimizing radiation-induced lung injury after concurrent chemoradiotherapy has become an increasingly important clinical consideration. In a secondary analysis of RTOG 0617, the incidence of grade 3 or higher pneumonitis was lower with IMRT than with 3D-CRT, despite the IMRT cohort having larger PTVs and higher lung V_5Gy_ (where V_XGy_ indicates the percentage of volume receiving at least X Gy), while lung V_20 Gy_ was similar between the groups [57]. These findings have supported the increasing adoption of IMRT for chemoradiotherapy in unresectable stage III NSCLC. In Japan, the use of IMRT expanded during the late 2010s and early 2020s, and its application to lung cancer has increased in parallel with the introduction of maintenance durvalumab following chemoradiotherapy [58, 59].

In addition, osimertinib in the LAURA trial has been associated with cardiac adverse events, including arrhythmia, deterioration of cardiac function, and cardiac arrest. As patients with EGFR-mutated NSCLC may achieve long-term survival, radiation-induced cardiac toxicity—previously a major concern primarily among long-term survivors of breast cancer or lymphoma receiving thoracic radiotherapy—has become an increasingly relevant issue in this population. According to the cardio-oncology guidelines of the European Society of Cardiology, the risk of radiation-induced cardiac toxicity is categorized based on the mean heart dose: < 5 Gy is considered low risk, 5–15 Gy moderate risk, 15–25 Gy high risk, and ≥ 25 Gy very high risk [60]. Assessment of baseline cardiovascular risk before thoracic radiotherapy is therefore recommended, followed by risk-adapted cardiovascular monitoring. In high-risk cases, collaboration with cardiologists is essential to optimize long-term management. Consequently, there is growing emphasis on reducing not only lung dose but also cardiac dose during thoracic radiotherapy. Techniques such as cardiac-sparing VMAT and particle radiotherapy may help reduce both pulmonary and cardiac toxicities [61, 62]. Regarding particle radiotherapy, long-term outcomes of concurrent proton beam therapy and chemotherapy have shown a 5-year OS of 38.8%, with grade 3 pneumonitis in 8.9% of patients, no grade 3 cardiac events, and no grade 4 or higher adverse events [63]. A Japanese nationwide multi-institutional cohort also showed a low incidence of severe toxicity, with grade ≥ 3 pneumonitis in 1.3% of patients and no grade 4 or higher toxicities [64]. Thus, particle radiotherapy is expected to be a promising modality for reducing treatment-related toxicity in selected patients with stage III NSCLC. In addition, functional avoidance radiotherapy, guided by functional imaging such as ventilation maps, has emerged as a promising strategy to further minimize damage to critical lung regions [65–67]. The lung and heart are also lymphocyte-rich organs, and irradiation of these structures may contribute to treatment-related lymphopenia. A secondary analysis of RTOG 0617 showed that the effective radiation doses to immune cells (EDIC), estimated mainly from the mean lung dose, mean heart dose, was significantly associated with worse OS [68]. A similar association has also been reported in the durvalumab era [69]. Therefore, lymphocyte-sparing radiotherapy has recently been proposed to preserve immune function, particularly when radiotherapy is combined with immunotherapy [70]. With the increasing complexity of treatment planning and the need to optimize dose distribution across multiple organs at risk, artificial intelligence-based planning approaches may play an important role in treatment optimization.

In stage III NSCLC, the introduction of ICIs has led to a rapid accumulation of new evidence, and treatment strategies are evolving quickly in both resectable and unresectable disease. At the same time, the boundary between these categories has become increasingly blurred. Technical resectability does not necessarily imply that surgical resection is oncologically recommended. In this rapidly evolving landscape, radiation oncologists should not regard the assessment of resectability as solely the surgeon’s responsibility. Rather, it is essential to evaluate whether the disease is oncologically suitable for surgical resection, identify patients in whom radiotherapy-based approaches may be more appropriate, and participate as equal partners with thoracic surgeons and medical oncologists in multidisciplinary decision-making based on the most up-to-date evidence.

## Oligometastatic stage IV

### Key clinical evidence and future directions

The concept of “oligometastasis” has been proposed as an intermediate state between localized and widespread metastatic disease. Recently, consensus recommendations for its characterization and classification have been proposed [71, 72]. A key study that popularized this concept was the SABR-COMET trial, a randomized phase II study comparing standard-of-care (SOC) treatment alone with SOC plus SBRT in patients with one to five oligometastases. The addition of SBRT significantly improved OS (HR, 0.50; 95% CI 0.30–0.84; *p* = 0.008) [73]. However, the SABR-COMET trial included multiple primary tumor types, and approximately 20% of enrolled patients had lung cancer. Subsequently, with growing interest in the potential role of local consolidative therapy (LCT) for oligometastatic disease, clinical trials have increasingly focused specifically on oligometastatic stage IV NSCLC.

Representative phase II–III trials are summarized in Table 3. Gomez et al. conducted a randomized phase II trial comparing systemic therapy plus LCT, including radiotherapy and/or surgery, with systemic therapy alone in patients with NSCLC who had a controlled primary tumor and up to three metastatic lesions [74, 75]. Radiotherapy was delivered in 96% of patients in the LCT group. Although the incidence of grade 3 or higher adverse events was higher in the LCT group, both PFS and OS were significantly improved. Similarly, Iyengar et al. evaluated patients with EGFR-wild-type and ALK-negative NSCLC with up to five oligometastases, comparing chemotherapy alone with chemotherapy plus SBRT or hypofractionated radiotherapy [76]. The addition of SBRT or hypofractionated radiotherapy significantly improved PFS, although grade 3 or higher adverse events were more frequent in the SBRT or hypofractionated radiotherapy arm. Since these studies, several additional phase II trials have been reported [77–83]. However, substantial heterogeneity exists across studies in terms of the definition of oligometastasis, the molecular characteristics of NSCLC, and the patterns and timing of LCT. Consequently, the reported clinical outcomes have varied considerably.

**Table 3:** Representative phase II–III trials of oligometastatic stage IV NSCLC

Trial/author (year)	Phase	*N*	Eligibility	Intervention	mOS (months)	mPFS (months)	AEs ≥ G3 (%)
Gomez DR, et al. (2016) [70, 71]	RII	49	Induced oligopersistence ≤ 3 metastases ± primary NSCLC	SBRT, hypofractionated RT, chemoradiotherapy, and/or surgery followed by SOC versus SOC	41.2 versus 17.0	14.2 versus 4.4	20.0 versus 16.6
Iyengar P, et al. (2018) [72]	RII	29	Induced oligopersistence ≤ 5 metastases ± primary; ≤ 3 in the liver or lung EGFR-wt and ALK-negative NSCLC	SBRT or hypofractionated RT followed by SOC versus SOC	NR versus 17	9.7 versus 3.5	28.6 versus 20
CURB (2024) [73]	RII	59*	Induced oligoprogression ≤ 5 metastases including primary NSCLC and breast cancer	SBRT + SOC versus SOC	–	10.0 versus 2.2*	62 versus 41 (≥ G2)
STOP (2025) [74]	RII	40*	Induced oligoprogression ≤ 5 metastases including primary NSCLC and other malignancies	SBRT + SOC versus SOC	31.2 versus 27.4	8.4 versus 4.3	3.4 versus 0
CURE-OLIGO (2025) [75]	II	19	Synchronous oligometastases ≤ 3 metastases; ≤ 2 organs EGFR-wt and ALK-negative NSCLC	Chemoradiotherapy for thoracic lesions + LCT for distant metastases	42.1	8.6	≥ 74 (neutropenia)
ETOP CHESS (2025) [76]	II	49	Synchronous oligometastases ≤ 3 metastases EGFR-wt and ALK-negative NSCLC	Chemotherapy, ICI, and SBRT for distant metastases, RT or surgery for the primary lesion, and maintenance ICI	74.9% (1-year)	33% (1-year)	34
Chen H, et al. (2025) [77]	II	59	Induced oligopersistence ≤ 5 metastases including primary; ≤ 3 organs EGFR-wt and ALK/ROS1-negative NSCLC	SBRT or stereotactic RT for all lesions followed by maintenance systemic therapy	88.9% (2-year)	29	22.0
Li Q, et al. (2025) [78]	II	35	Synchronous oligometastasis ≤ 5 metastases; ≤ 3 organs EGFR-wt and ALK/ROS1-negative NSCLC	Chemotherapy + ICI followed by maintenance ICI + SBRT ± RT for distant metastases	67.7% (2-year)	10.4	57.1
Ghafoor A, et al. (2025) [79]	II	37	Induced oligoprogression/recurrence after prior TKI^†^ or synchronous oligometastasis EGFR-mutated NSCLC	TKI^†^ followed by LCT at oligoprogression with subsequent TKI^†^, or upfront LCT followed by TKI^†^	–	11.9	≥ 14 (pain)
SINDAS (2023) [80]	RIII	133	Synchronous oligometastasis ≤ 5 metastases; ≤ 2 lesions in 1 organ EGFR-mutated NSCLC	TKI^‡^ + RT for distant metastases, the primary tumor, and involved regional nodes versus TKI^‡^	25.5 versus 17.4	20.2 versus 12.5	5.9 versus 1.5 (pneumonitis)
NROG-002 (2025) [81]	RIII	118	Synchronous oligometastasis or induced oligoprogression/persistence ≤ 3 organs EGFR-mutated NSCLC	TKI^§^ + thoracic RT for the primary, and involved regional nodes versus TKI^§^	34.4 versus 26.2	17.1 versus 10.6	11.9 versus 5.1

ALK, Anaplastic lymphoma kinase fusions; EGFR, Epidermal growth factor receptor; ICI, Immune checkpoint inhibitor; LCT, Local consolidative therapy; mOS, Median overall survival; mPFS, Median progression-free survival; NR, Not reached; R, Randomized; ROS1, ROS proto-oncogene 1; RT, Radiotherapy; SOC, Standard of care; TKI, Tyrosine kinase inhibitor; wt, Wild-type. Other abbreviations are the same as Tables 1 and 2

*NSCLC subgroup

^†^Osimertinib

^‡^Gefitinib, erlotinib, or icotinib

^§^Icotinib

From a molecular perspective, the available evidence suggests that LCT may be particularly beneficial in patients with EGFR-mutated oligometastatic NSCLC. Two phase III trials conducted in China specifically evaluated this population and both demonstrated improved OS and PFS with the addition of radiotherapy. The SINDAS trial, which enrolled patients with synchronous oligometastases (≤ 5 metastases with ≤ 2 lesions in any single organ), compared EGFR-TKI alone with EGFR-TKI combined with radiotherapy delivered to all metastatic sites as well as the primary tumor or involved regional lymph nodes [84]. Similarly, the NROG-002 trial evaluated patients with oligo-organ metastases and compared first-line concurrent EGFR-TKIs and thoracic radiotherapy with EGFR-TKIs alone [85]. In contrast, comparable benefits have not been consistently observed in molecularly unselected populations. In the NRG-LU002 randomized phase II/III trial, patients with ≤ 3 extracranial oligometastatic sites who had not progressed after first-line systemic therapy were randomized to LCT, including radiotherapy and/or surgery, followed by maintenance systemic therapy, or to maintenance systemic therapy alone. At the planned interim analysis, the trial was closed for futility, as the addition of LCT did not improve survival and was associated with increased toxicity [86]. Similarly, JCOG 2108, a randomized phase III study comparing systemic chemotherapy followed by maintenance therapy with or without local consolidation therapy for postoperative oligometastatic recurrent NSCLC, was also terminated early because of slow accrual [87]. Collectively, these findings suggest that certain molecularly defined subgroups may derive greater benefit from combined systemic therapy and LCT, although confirmation in broader patient populations is still required.

Overall, while accumulating evidence indicates that local radiotherapy may contribute to improved overall survival in selected patients with oligometastatic NSCLC, the substantial heterogeneity in patient characteristics, metastatic burden, and treatment approaches currently precludes the establishment of a definitive treatment strategy. Further efforts to refine patient selection and generate robust prospective evidence remain essential.

## Conclusions

Radiotherapy has become an integral component of the multidisciplinary management of NSCLC, supported by advances in high-precision irradiation techniques that enable the delivery of ablative doses while minimizing treatment-related toxicity. At the same time, the therapeutic landscape of NSCLC has rapidly evolved with the introduction of ICIs, targeted therapies, and advances in surgical procedures. These developments have reshaped the clinical role of radiotherapy across disease stages—from SBRT and particle therapy for early-stage disease, to its integration with systemic therapy in locally advanced disease, and to its emerging role in oligometastatic settings. However, optimal patient selection, treatment sequencing, and integration with modern systemic therapies remain areas of active investigation. As treatment strategies continue to evolve, multidisciplinary collaboration among radiation oncologists, thoracic surgeons, and medical oncologists is increasingly essential. A deeper understanding of the current evidence and ongoing clinical trials will be critical to further refine the role of radiotherapy and maximize its contribution to improving outcomes for patients with NSCLC.
